# Differential Gene Expression Responding to Low Phosphate Stress in Leaves and Roots of Maize by cDNA-SRAP

**DOI:** 10.1155/2020/8420151

**Published:** 2020-07-20

**Authors:** Lei Yan, Liang Su, Rui Li, Hao Li, Jianrong Bai, Fengjie Sun

**Affiliations:** ^1^College of Agriculture, Shanxi Agricultural University, Taiyuan, 030031 Shanxi, China; ^2^Collee of Food Engineering, Jilin Engineering Normal University, Changchun, 130052 Jilin, China; ^3^School of Science and Technology, Georgia Gwinnett College, Lawrenceville, GA 30043, USA

## Abstract

Phosphate (Pi) deficiency in soil can have severe impacts on the growth, development, and production of maize worldwide. In this study, a cDNA-sequence-related amplified polymorphism (cDNA-SRAP) transcript profiling technique was used to evaluate the gene expression in leaves and roots of maize under Pi stress for seven days. A total of 2494 differentially expressed fragments (DEFs) were identified in response to Pi starvation with 1202 and 1292 DEFs in leaves and roots, respectively, using a total of 60 primer pairs in the cDNA-SRAP analysis. These DEFs were categorized into 13 differential gene expression patterns. Results of sequencing and functional analysis showed that 63 DEFs (33 in leaves and 30 in roots) were annotated to a total of 54 genes involved in diverse groups of biological pathways, including metabolism, photosynthesis, signal transduction, transcription, transport, cellular processes, genetic information, and organismal system. This study demonstrated that (1) the cDNA-SRAP transcriptomic profiling technique is a powerful method to analyze differential gene expression in maize showing advantageous features among several transcriptomic methods; (2) maize undergoes a complex adaptive process in response to low Pi stress; and (3) a total of seven differentially expressed genes were identified in response to low Pi stress in leaves or roots of maize and could be used in the genetic modification of maize.

## 1. Introduction

Maize is a well-known economically important crop in the world. However, the maize production is commonly affected by phosphate (Pi) deficiency in soil due to the high percentage of fixed Pi that cannot be absorbed by plants [1]. Plants have evolved many adaptive strategies to cope with low Pi supply with many genes involved in the response to Pi starvation [2]. For example, the Pi uptake is increased by directly inducing the expression of Pi transporters, while the inositol polyphosphates are shown to play important roles in the intracellular Pi signaling [3–5]. In order to understand the molecular mechanisms responding to low Pi stress in maize, it is becoming increasingly important to characterize the transcriptional changes and signal transduction pathways in plants under low Pi stress.

Previous studies have investigated gene expression patterns in response to plant defense against low Pi [6–12]. For example, microRNAs (e.g., *miRNA399b* and *Zma-miR3*) are induced to express in the roots of maize under low Pi stress [12], while studies of metabolite profiling and genome-wide association have shown that the gene encoding glucose-6-phosphate-1-epimerase is capable of correlating traits based on the Pi levels in maize seedlings [9]. Furthermore, abscisic acid (ABA) plays a dominant role in regulating candidate genes in response to Pi stress in *Arabidopsis* [11].

The initial response to Pi starvation in plants includes the sensation and communication of the amount of Pi at both the cellular and molecular levels in various organs (e.g., roots and leaves) with the roots establishing the initial response to low Pi stress [2]. Studies show that Pi starvation promotes growth of primary roots but reduces the numbers of lateral roots and lateral root primordia, while the Pi intake under Pi stress is enhanced by greater lateral root branching density [13–15]. Transcriptomic analyses of maize gene expression under Pi stress have been carried out in maize roots [6, 7, 11, 13]. These studies showed that auxin signaling is involved in the response to Pi stress, while the lateral root development is regulated by DNA replication, transcription, protein synthesis and degradation, and cell growth [13].

Plant survival under Pi stress depends on the tolerance at the whole-plant level rather than individual tissues or organs because low Pi usually leads to detrimental effects on the entire plant. For example, studies have shown the accumulations of the flavonoid pigment anthocyanin, di- and trisaccharides, and metabolites of ammonium metabolism in leaves under low Pi stress [6, 16]. A total of 78 differentially expressed fragments (DEFs) in leaves and roots of maize have been identified using the cDNA-amplified fragment length polymorphism (cDNA-AFLP) technique [7], while the induction of acid phosphatase and activities of catalase/peroxidase in both stems and roots of Pi-tolerant cultivar of maize under Pi stress is identified much earlier than that in the Pi-sensitive cultivar [6]. Recent studies have shown that the nucleic acids, organic acids, and sugars were increased, while the phosphorylated metabolites, certain amino acids, lipid metabolites, and nitrogenous compounds were decreased in maize seedlings under low Pi stress [9]. Furthermore, it has been reported that the peroxidase and superoxide dismutase-related genes and the lipid peroxidation genes were upregulated in maize leaves [17]. Therefore, simultaneous analyses of gene expression patterns in response to Pi stress in multiple plant organs, e.g., roots and leaves, provide more accurate understanding of the molecular mechanisms of Pi tolerance in maize.

This study focused on the gene expression in both roots and leaves of a maize inbred line in response to Pi stress using the sequence-related amplified polymorphism (SRAP) technique [18]. Due to its advantages of being easy, effective, and fast, the SRAP analysis has been widely used to construct a genetic map and to investigate molecular diversity and comparative genomics in various species of crops [18–22]. Besides the genomic DNA, cDNA can also be used as templates to identify the fragment length polymorphism using SRAP primers. For example, the cDNA-SRAP technique has been utilized successfully to study the differential gene expression in several species of plants [22–26]. In our study, we used this simple and effective method with low cost to study the gene expression patterns in both roots and leaves of maize under low Pi stress. Our goals were to (1) identify the DEFs that are transcriptionally regulated in response to Pi stress in maize and (2) to further identify the candidate genes of maize involved in response to low Pi stress.

## 2. Materials and Methods

### 2.1. Plant Material and Growth Conditions

The previously isolated maize inbred line 478 showing high efficiency of intaking Pi was used in this study [27]. Seeds were surface-sterilized with 75% ethanol for 30 sec, rinsed three times with sterile distilled water, and planted in boxes filled with sterilized perlite. A total of 40 3-day-old seedlings were transferred to a continuously aerated nutrient solution with high Pi (HP) content (1 mM KH_2_PO_4_) for 7 days. Then, 20 of the seedlings were transferred into the low Pi (LP) solution (0.01 mM KH_2_PO_4_)), while the other 20 seedlings were maintained in the HP solution (as the control group). The basal nutrient solution contained 2 mM Ca(NO_3_)_2_, 0.65 mM MgSO_4_, 25 *μ*M Fe-EDTA, 5 *μ*M MnSO_4_, 50 *μ*M KCl, 2 *μ*M ZnSO_4_, 0.5 *μ*M CuSO_4_, 0.005 *μ*M (NH_4_)_6_Mo_24_, and 25 m*Μ* H_3_BO_4_. In addition, K^+^ was supplied in the LP solution in the form of 0.99 mM KCl. The seedlings were kept in an artificial climate chamber under a photoperiod of 8 h of darkness (18°C) and 16 h of light (28°C; 100 *μ*mol m^−2^ s^−1^ photon flux density). Humidity was maintained at ~70%.

### 2.2. Sample Collection and RNA Preparation

Samples of leaves and roots were harvested at 0 day from the HP solution and at 3, 5, and 7 days from the LP solution, respectively. These eight samples were frozen immediately using liquid nitrogen and stored at -80°C for further analysis. Leaf or root tissues were ground to fine powder in liquid nitrogen to extract the total RNA with Trizol Reagent (Sangon Biotech, Shanghai, China) according to the manufacturer's instructions.

### 2.3. cDNA-SRAP Analysis

Synthesis of the first cDNA strand was performed with M-MLV Reverse Transcriptase (Promega). Each PCR reaction mixture (20 *μ*L) of the cDNA-SRAP amplification contained 140 ng of cDNA, 0.25 mmol/L of dNTP mixture, 1.8 mmol/L of Mg^2+^, 0.5 *μ*mol/L of forward and reverse primers, respectively, and 1.0 U of Taq DNA polymerase (Takara) [28]. In order to reduce the amplifications of nonspecific fragments in the cDNA-SRAP analysis, the conditions of PCR amplification were optimized to be 35 cycles of PCR amplification with a 20-fold dilution of DNA template. Further analyses were based on a total of 60 pairs of SRAP primers showing reliable amplifications indicated by rich and strong bands (Table 1). The SRAP thermal cycling using PCR was as follows: an initial predenaturation at 94°C for 5 min, followed by five cycles of denaturation at 94°C for 30 sec, annealing at 35°C for 30 sec, and extension at 72°C for 1 min. Then, the annealing temperature was increased to 50°C for another 35 cycles with a terminal extension at 72°C for 10 min. The PCR products were separated using 6% urea-polyacrylamide gel electrophoresis (PAGE) and examined using the silver-staining method. For each primer combination, the final PCR products from a series of days (i.e., 0, 3, 5, and 7) of treatments of Pi stress were loaded in order into lanes next to each other in the PAGE gel for the comparison of band density for bands of the same size.

### 2.4. Identification, Isolation, and Sequencing of Differentially Expressed Fragments (DEFs)

The slices of the PAGE gel containing the target bands showing high expression, over 200 bp in length, and representing one of the identified differential expression patterns were excised and then hydrated in 50 *μ*L of Tris-EDTA buffer (pH 8.0) and incubated overnight at 37°C. The eluted fragments were reamplified using PCR with the same primers and the same conditions as those for the cDNA-SRAP analysis. The reamplified products were cloned using the pMD18-T vector (Takara) and then sequenced. These sequences of the transcript-derived fragments were compared to the protein database using the BLASTx algorithm at the National Center of Biotechnology Information (https://blast.ncbi.nlm.nih.gov/Blast.cgi). These sequences were also annotated using the Kyoto Encyclopedia of Genes and Genomes (KEGG) database (https://www.genome.jp/kegg/) to reveal the biochemical and physiological pathways.

### 2.5. Relative Quantitative Real-Time PCR (qRT-PCR) Analysis

To further verify the results of the cDNA-SRAP analysis, the cDNAs derived from the eight leaf and root samples of maize were randomly selected to quantitatively examine the expression levels of selected fragments using the qRT-PCR. The amplification mixture contained 1 *μ*L of cDNA, 10 *μ*L of 2x SYBR® Premix Ex Taq™ II (Perfect Real Time) (Takara), and 0.5 *μ*mol/L forward and reverse primers, respectively. The thermal cycling protocol consisted of preincubation at 95°C for 3 min, followed by 40 cycles of denaturation at 95°C for 10 s, and annealing at 60°C for 20 s. A standard melting curve was generated at the end of the amplification with the measurements recorded between 60°C and 95°C used to calculate the PCR efficiency (*E*). The qRT-PCR amplifications were conducted in parallel in triplicate with the normalization performed using the transcript level of the constitutively expressed *Tubulin* gene as control in all samples. Relative expression ratios were calculated according to Advanced Relative Quantification of Roche LightCycler 480 software release 1.5.0.

## 3. Results and Discussion

### 3.1. cDNA-SRAP Analysis

Investigations of the transcriptional changes and signal transduction pathways of genes are important for understanding the molecular mechanisms underlying the response to the low Pi stress in maize. Commonly used transcriptomic techniques include microarray [29], RNA sequencing [30], cDNA-AFLP [31], and cDNA-SRAP [18], in addition to the differential display RT-PCR [32], which was superseded by microarray methods [33]. Each of these methods showed various advantages and disadvantages. Although the high-cost microarray method can accurately detect highly expressed genes, it is difficult to detect genes with low abundance because a strong hybridization background and erroneous annotations may occur because of the limited data in the available databases. RNA sequencing is simple and efficient, but disadvantageous due to its being costly for high coverage of transcripts, inaccurate detection of genes with low abundance, and lack of the visual display of the gene expression patterns. The cDNA-AFLP provides a direct, efficient, sensitive, and reproducible approach for the identification of novel genes in plants, while not requiring prior information of gene sequences and is therefore a favored tool used commonly to identify differentially expressed genes (DEGs). However, the disadvantages of cDNA-AFLP analysis include its high cost, the requirement of the double-enzyme digestions of the cDNA, and the difficult optimization of the experimental conditions. Among these transcriptomic methods, the cDNA-SRAP is advantageous due to its low cost, simple and efficient operation, high stability and repeatability, and high coverage of transcripts.

In comparison to other transcriptomic techniques, the cDNA-SRAP method has been widely used to study the differential gene expression because it does not have the disadvantages of the other methods and it has several unique advantages. First, the cDNA-SRAP analysis is a valuable tool for high-throughput gene expression analysis because of its generation of reliable and repeatable gene expression data and identification of a high coverage of transcript tags. Second, in contrast to the hybridization-based techniques, the cDNA-SRAP method allows a more thorough and comprehensive gene expression analysis at a large scale because of its capability of direct, simultaneous comparison of gene expression levels in different organs or tissues at different developmental stages under the treatment of various types of stress investigations. Third, in comparison to the cDNA-AFLP method, the cDNA-SRAP analysis costs less but is easier to operate because it does not require double-enzyme digestion of cDNA. The effectiveness of the cDNA-SRAP method is demonstrated evidently in our study showing the annotations of the cDNA fragments identified in both roots and leaves of maize to the diverse classes of known genes.

### 3.2. Expression Patterns of DEFs Revealed by cDNA-SRAP

The cDNA-SRAP transcript profiling in both roots and leaves of maize under low Pi stress over 0, 3, 5, and 7 days was performed using 60 primer pairs to investigate the gene expression patterns in response to low Pi stress in maize. The sizes of a total of 2571 DNA fragments that we revealed ranged from 80 to 800 bp with an average of ~43 fragments obtained per primer pair. A total of 2494 identified fragments (97%) were identified as DEFs with an average of ~42 DEFs obtained per primer pair. These DEFs were categorized into 13 classes of differential expression patterns, while the nondifferentially expressed fragments were grouped in Class 14 (Table 2). These 14 classes of expression patterns include (1) transient-induced expression showing expressions only at either 3-, 5-, or 7-day treatment of low Pi (LP); (2) rapidly switched-off expression in LP but with expression in high Pi (HP) treatment; (3) gradually switched-off expression showing expression in HP and 3- or both 3- and 5-day treatments of LP then switched off in both 5- and 7-day or in 7-day treatment of LP, respectively; (4) induced expression induced in 3 or 5 days and then expressed continuously in LP; (5) switched-off–induced expression in HP but switched off in 3- or in 3- and 5-day treatments of LP then expressed continuously in LP; (6) induced–switched-off–induced expression in 3- and 5-day treatments of LP but switched off in HP and 5-day treatment of LP; (7) down expression showing expression in HP but switched off in LP; (8) down-up expression showing high expression in HP but continuous down expression in LP; (9) down-up-down expression showing high expression in HP and 5-day treatment of LP but down expression in 3- and 7-day treatments of LP; (10) up expression in HP then up expressed continuously in LP; (11) up-down-up expression showing expression in HP then up expression in 3-day, down expression in 5-day, and up expression in 7-day treatments of LP; (11) up-down expression showing expression in HP then up expression in 3-day and down expression in both 5- and 7-day treatments of LP; (12) up-down-up expression showing expression in HP and 5-day treatment of LP with higher expression in 3- and 7-day treatments of LP; (13) induced–switched-off expression showing expression in 3- and 5-day treatments of LP then switched off in HP and 7-day treatment of LP; and (14) constitutive expression showing high nondifferential expression in both HP and LP.

In leaves, there were a total of 994 DEFs (~80%) revealed in Class 1-5 expression patterns with each class containing more than 80 DEFs. Class 13 contained 67 DEFs, while less than 50 DEFs were identified in each of the other eight classes. Similar distributions of DEFs were identified in roots as those in leaves. A total of 960 DEFs (~72%) were identified in four classes (1, 2, 4, and 5) of expression patterns with each class containing more than 90 DEFs, while less than 60 DEFs were categorized in each of the other nine classes.

Studies using other transcriptomic analyses to investigate the response of maize to low Pi stress identified varied numbers of DEFs. For example, microarray analysis of the lateral root primordium zone responding to low Pi showed that in 2 days of treatment, a total of 148 differentially expressed transcripts contained 71 upregulated and 77 downregulated transcripts, and in 8 days of low Pi treatment, a total of 549 DEGs contained 270 upregulated and 279 downregulated genes in the roots of maize [13]. Studies using strand-specific RNA-Seq transcriptomic analyses of leaves and roots of low P-tolerant and P-sensitive maize inbred lines identified a total of 5900 DEFs in the low-P-sensitive and 3389 DEFs in the low-P-tolerant maize [6]. A total of 142 DEFs with 121 upregulated and 21 downregulated in LP were detected using the cDNA-AFLP method with 136 primer pairs to screen LP-tolerant maize under HP and LP stress conditions [7]. In comparison to our results, these studies suggested that cDNA-SRAP analysis is more powerful and advantageous than other transcriptomic analyses in identifying the number of DEFs in maize responding to low Pi stress, while our results provide a more complete and integrated variations at the transcriptional level between the treatment of HP and LP stress.

### 3.3. Sequence Analysis and Functional Annotation

A total of 63 DEFs (33 in leaves and 30 in roots) representing 11 out of the 13 classes of differential expression patterns with high expression and longer than 200 bp in length were selected and isolated from the polyacrylamide gels, reamplified by PCR, and sequenced (GenBank accession numbers JZ983140–JZ983202). The sequences of the 63 DEFs were annotated based on the GenBank protein database at the National Center of Biotechnology Information (https://blast.ncbi.nlm.nih.gov/Blast.cgi) using the BLASTx algorithm (Table 3). Significant homology (*E*value: 0.001) was revealed for 60 DEFs (~95%) to genes with known functions, while 3 DEFs (~5%) were annotated to genes without allocated functions. There were a total of four genes each annotated by 2 or 3 DEFs, including putative non-LTR retroelement reverse transcriptase (DEFs 292 of induced and 293 of up expression) and rRNA intron-encoded homing endonuclease (DEFs 267 of induced and 268 of up-down-up expression) in roots and bZIP transcription factor ABI5 (DEFs 093 of transient-induced (5 day), 094 of gradually switched-off, and 096 of transient-induced (3 day) expression), and tubulin gamma complex-associated protein (DEFs 112 and 114 of up-down expression) in leaves. It was worth noting that different DEFs of each one of these four genes showed compatible rather than conflicting expression patterns, probably because these genes were not detected due to either having a low expression or containing multiple copies of transcripts, suggesting that the different expression patterns existed for different organs (e.g., roots or leaves) under various treatments (i.e., the length of treatments under low Pi stress) in relation to the distance from the sites of treatments, e.g., the quick response in roots, while the delayed response in leaves.

**Figure 1:**
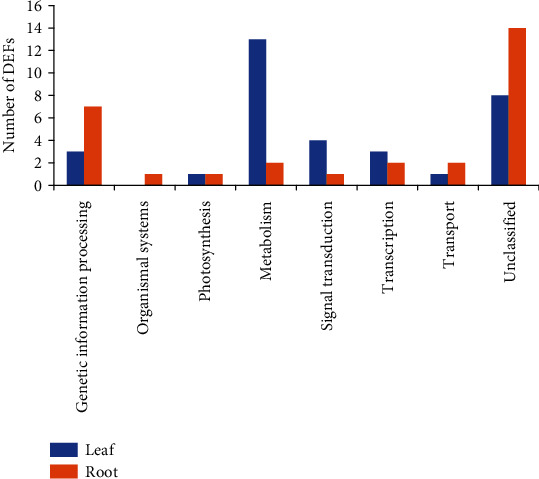
Functional annotations of 63 DEFs in roots and leaves of maize under low Pi stress based on the KEGG database.

These 63 DEFs in maize under low Pi stress were further annotated into eight metabolic pathways with a group of “unclassified” based on the Kyoto Encyclopedia of Genes and Genomes (KEGG) database (https://www.genome.jp/kegg/). The “unclassified” group contained the largest number of DEFs (~35%), followed by the group of “metabolism” (~24%) containing proteins of unknown functions and hypothetical or unclassified proteins (Figure 1). The lowest numbers of DEFs (~1.5%) were revealed in three pathways including “cellular processes,” “organismal systems,” and “photosynthesis.” These results indicated that the identified DEFs were involved in diverse groups of biological pathways in response to low Pi stress in maize. It was noted that the varied numbers of DEFs were revealed in different pathways in leaves and roots, suggesting that there are alternative molecular mechanisms responding to low Pi stress in leaves and roots of maize. Furthermore, previous studies of the response of maize to low Pi stress have showed that the pathway of “metabolism” contained the most number of DEFs [7, 8, 13], suggesting that “metabolism” is likely one of the most important pathways involved in response to low Pi stress in both roots and leaves of maize. These results further indicate that among these identified pathways, “signal transduction” and “genetic information processing” may also play important roles in response to low Pi stress in leaves and roots of maize, respectively. To summarize, many biological pathways are involved in the response to low Pi stress in maize, ultimately affecting its development, growth, and production; therefore, it is important to investigate comprehensively the molecular mechanisms responding to the low Pi stress in maize.

### 3.4. DEFs in Response to Low Pi Stress in Maize

Although roots are the first organs in plants to respond to low Pi, plant survival depends on whole-plant tolerance to low Pi stress. Therefore, it is important to investigate both the root and the leaf in order to achieve a comprehensive understanding of the molecular mechanisms responding to low Pi stress in plants. Previous studies detected the total number of DEFs in maize under low Pi stress without any differentiation between the numbers of DEFs identified specifically from either leaves or roots [7, 8, 13]. Furthermore, tissue-specific gene expression patterns under low Pi conditions were revealed. For example, genes in auxin biosynthesis and signaling and cell defense response protein degradation were upregulated and the expression of genes involved in cell proliferation and growth decreased, while the meristem region and other tissues related to genes not differentially expressed in the lateral root primordium zone of the primary root [13].

Our results showed that there were more DEFs in roots than those in leaves in seven out of the 13 classes of DEFs, including the transient-induced (both 5 and 7 days), induced, switched-off–induced, induced–switched-off–induced, down, up, and up-down-up expression patterns. More DEFs was also realized in roots than those in leaves showing the constitutive expression pattern. Comparative proteomic analyses of Pi responses in the roots of maize revealed that pathways of citrate secretion, sugar metabolism, and root-cell proliferation played important roles in enhancing tolerance to low Pi conditions [8]. In comparison to both *Arabidopsis thaliana* and rice, maize showed different responsive patterns to LP stress; specifically, the growth of primary roots was promoted, and the formation of lateral roots was inhibited in maize [13]. Using the cDNA-AFLP analysis, Jiang et al. [7] identified a total of 78 DEFs and 9 genes in maize highly homologous to genes in both *Arabidopsis thaliana* and *Oryza sativa* involved in phosphorus metabolism, including phosphorus circulation, transportation, or response. Our results showed that there were more DEFs showing induced than down expressions in roots, while more DEFs were identified with transient-induced, rapidly switched-off, and induced expressions in roots than those in leaves, indicating that roots may respond more strongly to low Pi stress than leaves at transcriptional level.

### 3.5. Quantification of DEF Expression by qRT-PCR

**Figure 2:**
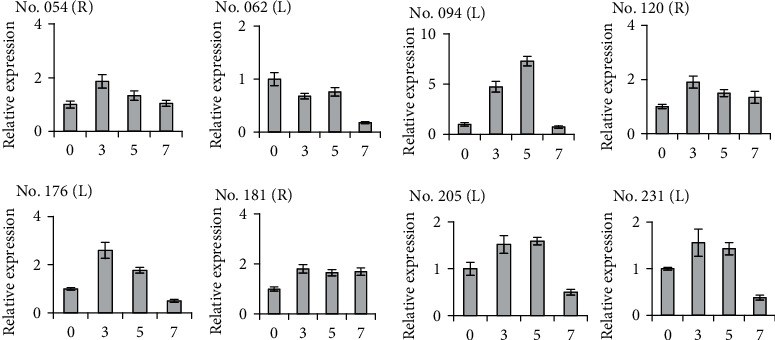
The qRT-PCR analysis of 8 DEFs revealed by cDNA-SRAP method in leaves (L) and roots (R) of maize in a series of days (0, 3, 5, and 7) of treatments under low Pi stress.

To verify the results of cDNA-SRAP, qRT-PCR analysis was used to further quantify the expression of 8 DEFs (3 of roots and 5 of leaves) randomly selected from the 63 DEFs revealed by cDNA-SRAP analysis sampled at 0, 3, 5, and 7 days in the treatment of low Pi stress (Figure 2). Results of the qRT-PCR analysis showed that there was no significant change in the transcript level of the endogenous reference gene *tublin*, validating the experimental conditions and qPCR analyses. The correlation coefficient (*R*^2^) for the *tublin* reference gene was 0.998, while the PCR efficiency (*E*) calculated from the slope of the standard melting curve was 105%. The *R*^2^ and *E* values for the 8 target gene transcripts varied from 0.991 to 1 and from 94% to 133%, respectively. The results of quantitative expression of these 8 DEFs were consistent with the expression patterns revealed by the cDNA-SRAP analysis (data not shown). For example, there were two bands on the PAGE gels of DEFs 120 and 181, suggesting that there might be two transcripts or homologous genes. This was confirmed by the DEF 181 annotating the vacuolar ATPase subunit H protein because there were indeed two vacuolar ATPase subunit H proteins in maize [34]. The verification of the cDNA-SRAP analysis provided by the qRT-PCR method strongly indicate that the cDNA-SRAP analysis is reliable, easy to operate, and cost efficient to study the differential gene expressions at a large scale in plants.

### 3.6. Candidate Genes in Response to Low Pi Stress in Maize

Our results showed that among the 54 genes annotated by the 63 DEFs (33 in leaves and 30 in roots), there were a total of seven genes found to be expressed differentially in both roots and leaves of maize under low Pi stress (Table 3). These genes were annotated in most of the biological pathways classified by the KEGG database, except for pathway “organismal systems.” Among these seven genes, the gene encoding the photosystem II protein H was induced in both roots and leaves of maize (i.e., induced expression in leaves and up-down expression in roots), while the other six genes were expressed in opposite expression patterns in leaves and in roots, suggesting different molecular mechanisms responding to the low Pi stress in leaves and roots, respectively.

These seven differentially expressed genes (DEGs) in roots and leaves of maize under low Pi stress annotated with known functions were considered as the candidate genes of maize involved in response to low Pi stress (Table 3). These candidate genes encode aspartic proteinaseo oryzasin-1 (annotated by DEFs 005 and 013 in the pathway of “signal transduction”), bZIP transcription factor ABI5 (annotated by DEFs 093, 094, 096, and 101 in the pathway of “transcription”), copia-type pol polyprotein (annotated by DEFs 018 and 243 as “unclassified”), photosystem II protein H (annotated by DEFs 115 and 139 in the pathway of “photosynthesis”), rRNA intron-encoded homing endonuclease (annotated by DEFs 262, 267, and 268 in the pathway of “genetic information processing”), vacuolar ATPase subunit H protein (annotated by DEFs 176 and 181 in the pathway of “transport”), and violaxanthin de-epoxidase-related (annotated by DEFs 231 and 235 in the pathway of “metabolism”). These DEFs showed different expression patterns in roots and leaves in response to low Pi stress. For example, the opposite expression patterns were observed for genes encoding vacuolar ATPase subunit H protein and violaxanthin de-epoxidase-related in roots and leaves. These results suggested that these candidate genes play important roles in response to low Pi stress in maize. No previous studies have reported the functions of these genes in response to low Pi stress in maize. Further function analysis of these candidate genes is necessary to help investigate the molecular mechanism of maize responding to the low Pi stress. It was noted that other genes identified by the cDNA-SRAP analysis were also candidate genes involved in the response to low Pi stress. Further studies were needed to characterize the roles these genes play in responding to Pi starvation. Here, we discuss the potential functions these genes play in response to low Pi stress.

The aspartic proteinase oryzasin-1 was expressed during seed ripening and germination in rice [35]. Aspartic protease in guard cell 1 (ASPG1) is suggested to be involved in ABA-dependent responsiveness and the overexpression of the ASPG1 gene can confer drought avoidance in *Arabidopsis* [36]. Therefore, we speculated that the aspartic proteinase oryzasin-1 is probably involved in ABA-dependent responsiveness to low Pi stress in maize.

Our results revealed for the first time that genes encoding photosystem II protein H showed differential expression patterns in maize under LP treatments. Previous studies identified DEGs related to photosynthesis with downregulation in maize under low Pi treatment [17], while our results indicated that the photosystem II protein H showed induced expression pattern in leaves and up-down regulation in root, suggesting that the photosystem II protein H may play an important role in photosynthesis in response to low Pi stress.

Previous studies identified the functions of the Pi-deficiency-induced long-noncoding RNA1 (PILNCR1) in the inhibitions of ZmmiR399-guided cleavage of ZmPHO2 [37]. We report for the first time that the rRNA intron-encoded homing endonuclease is involved in the response to the low Pi stress, while further investigations are needed to reveal the exact molecular mechanisms of how it participates in the response to low Pi stress in maize.

The vacuolar ATPase plays many key roles in plant growth and development and in stress response, while subunit H is vital to the activity and stability of the vacuolar ATPase [37, 38]. Two vacuolar ATPase subunit H protein genes are reported in maize (*ZmVHA-H1* and *ZmVHA-H2*) [34]. These genes contain *cis*-acting elements responsive to circadian control, abscisic acid, auxin, anaerobic, MeJA, and drought (*ZmVHA-H1*), as well as elements responsive to low-temperature, auxin, anaerobic, MeJA, drought, and wound (*ZmVHA-H2*). Studies have shown that overexpression of *ScVHA-H* in *Suaeda corniculata* improves the tolerance in transgenic alfalfa to salt and saline-alkali stresses [39]. These results suggest that the vacuolar ATPase subunit H protein may respond to low Pi stress by regulating the pH values in maize.

Studies have shown that the bZIP transcription factor ABI5 is involved in drought stress tolerance in barley [40], strawberry [41], *Arabidopsis* [42, 43], and other species of plants [44]. The main Pi response pathway (i.e., SIZ1-PHR1-miR399-PHO2) has been identified in *Arabidopsis* [45, 46], while SIZ1 negatively regulates the ABA signaling through sumoylation of ABI5 [47]. As a small ubiquitin-like modifier E3 ligase in plants, AtSIZ1 is identified as the focal controller of Pi starvation-dependent responses [46]. Furthermore, PHOSPHATE1 (PHO1) plays important roles in Pi homeostasis in *Arabidopsis* [48], while binding of ABI5 to the PHO1 promoter causes the downregulated expression of PHO1, which stops the ABA-insensitive germination of the ABI5 mutant [49]. These results suggest that ABI5 is involved in the response to low Pi stress by participating in either the SIZ1 or ABA signaling processes.

Violaxanthin de-epoxidase (VDE) is a type of lipocalin, which is characterized by a conserved structural organization with an eight-strand b-barrel and often binding hydrophobic molecules [50]. Studies have shown that VDEs play a key role in the pathway of carotenoid biosynthesis, which is involved in response to high light in bamboo [51], protecting the photosynthesis apparatus from damage caused by excessive light and chilling stress in tomato and *Arabidopsis* [52, 53], and improving tolerance to drought and salt stress in transgenic *Arabidopsis* [54]. Furthermore, studies showed that VDE1 in maize and teosinte share the conserved functions of VDE1 in other plants [55]. These results suggest that the VDE is probably involved in the response to low Pi stress in maize by participating in the pathway of photosynthesis.

## 4. Conclusions

We apply the cDNA-SRAP method to identify the DEFs in both roots and leaves of maize under low Pi stress. In comparison to other transcriptomic analyses, the cDNA-SRAP technique shows substantial advantages of being easy and effective with low cost in the analysis of differential gene expression in maize. A total of 13 classes of differential gene expression patterns are revealed with a total of 2494 DEFs (1202 in leaves and 1292 in roots) identified in response to Pi starvation in maize. Results of sequencing and functional analyses demonstrate that 63 DEFs are involved in diverse groups of biological pathways (i.e., metabolism, photosynthesis, signal transduction, transcription, transport, cellular processes, genetic information, and organismal system), suggesting evidently that maize undergoes a complex adaptive process in response to low Pi stress. We speculate that the eight candidate genes identified involved in response to low Pi stress could be used in the genetic modification of maize.

## Figures and Tables

**Table 1:** The forward (F) and reverse (R) primers used to make the 60 primer pairs used in the cDNA-SRAP analysis of roots and leaves of maize under low Pi stress. The 60 primer pairs include F1-R1, F1-R2, F1-R3, F1-R4, F1-R5, and F1-R9; F2-R2, F2-R3, F2-R4, and F2-R5; F4-R2, F4-R4, and F4-R5; F5-R1, F5-R2, F5-R3, F5-R4, and F5-R5; F6-R1, F6-R2, F6-R3, and F6-R4; F7-R1 and F7-R3; F8-R1, F8-R5, and F8-R4; F11-R11, F11-R12, F11-R13, F11-R14, and F11-R15; F13-R11, F13-R12, F13-R13, F13-R14, F13-R15, F13-R16, and F13-R19; F14-R16, F14-R17, F14-R18, and F14-R20; F15-R16, F15-R18, F15-R19, and F15-R20; F17-R16, F17-R17, F17-R18, F17-R19, and F17-R20; F19-R17, F19-R18, F19-R19, and F19-R20; F20-R16, F20-R17, F20-R19, and F20-R20.

Primers	Primer sequences 5′-3′
F1	TGAGTCCAAACCGGATA
F2	TGAGTCCAAACCGGAGC
F3	TGAGTCCAAACCGGAAT
F4	TGAGTCCAAACCGGACC
F5	TGAGTCCAAACCGGAAG
F6	TGAGTCCTTTCCGGTAA
F7	TGAGTCCTTTCCGGTCC
F8	TGAGTCCTTTCCGGTGC
F9	TGAGTCCAAACCGGTAG
F10	TGAGTCCAAACCGGTTG
F11	TGAGTCCAAACCGGTGT
F12	TGAGTCCAAACCGGTGA
F13	TGAGTCCAAACCGGCAT
F14	TGAGTCCAAACCGGTCT
F15	TGAGTCCAAACCGGAGG
F16	TGAGTCCAAACCGGAAA
F17	TGAGTCCAAACCGGAAC
F18	TGAGTCCAAACCGGTAA
F19	TGAGTCCAAACCGGTCC
F20	TGAGTCCAAACCGGTGC
R1	GACTGCGTACGAATTAAT
R2	GACTGCGTACGAATTTGC
R3	GACTGCGTACGAATTGAC
R4	GACTGCGTACGAATTTGA
R5	GACTGCGTACGAATTAAC
R6	GACTGCGTACGAATTGCA
R7	GACTGCGTACGAATTCAA
R8	GACTGCGTACGAATTCTG
R9	GACTGCGTACGAATTCGA
R10	GACTGCGTACGAATTCAG
R11	GACTGCGTACGAATTCCA
R12	GACTGCGTACGAATTATG
R13	GACTGCGTACGAATTAGC
R14	GACTGCGTACGAATTACG
R15	GACTGCGTACGAATTTAG
R16	GACTGCGTACGAATTTCG
R17	GACTGCGTACGAATTGTC
R18	GACTGCGTACGAATTGGT
R19	GACTGCGTACGAATTCGG
R20	GACTGCGTACGAATTGAT
R21	GACTGCGTACGAATTCCT
R22	GACTGCGTACGAATTCTT

**Table 2:** Characterization of differential expression patterns under Pi stress in maize. The four lanes on the PAGE gel show the amplification of DEFs in the order of 0-, 3-, 5-, and 7-day treatments of LP. The nondifferential expression pattern (Class 14) is included for comparison.

DEF expression pattern		Distribution of DEFs		
		Leaf	Root	Total
Class 1	Transient induced (3 days)	236 (9.2%)	168 (6.5%)	404 (15.7%)
	Transient induced (5 days)	118 (4.6%)	227 (8.8%)	345 (13.4%)
	Transient induced (7 days)	91 (3.5%)	175 (6.8%)	266 (10.3%)
Class 2	Rapidly switched off	235 (9.1%)	166 (6.5%)	401 (15.6%)
Class 3	Gradually switched off	144 (5.6%)	58 (2.3%)	202 (7.9%
Class 4	Induced	87 (3.4%)	95 (3.7%)	182 (7.1%)
Class 5	Switched off-induced	83 (3.2%)	129 (5.0%)	212 (8.2%)
Class 6	Induced-switched off-induced	25 (1.0%)	54 (2.1%)	79 (3.1%)
Class 7	Down	19 (0.7%)	28 (1.1%)	47 (1.8%)
Class 8	Down-up	13 (0.5%)	52 (2.0%)	65 (2.5%)
Class 9	Down-up-down	9 (0.4%)	8 (0.3%)	17 (0.7%)
Class 10	Up	23 (0.9%)	44 (1.7%)	67 (2.6%)
Class 11	Up-down	45 (1.8%)	29 (1.1%)	74 (2.9%)
Class 12	Up-down-up	13 (0.5%)	23 (0.9%)	36 (1.4%)
Class 13	Induced-switched off	67 (2.6%)	30 (1.2%)	97 (3.8%)
Class 14	Constitutive	33 (1.3%)	44 (1.7%)	77 (3.0%)
Total		1241 (48.3%)	1330 (51.7%)	2571 (100%)

**Table 3:** BLAST annotations of a total of 54 genes based on the 63 DEFs of leaves and roots in maize under low Pi stress categorized into 11 classes of differential expression patterns. The DEFs annotating the candidate genes in response to low Pi stress in maize are highlighted in bold.

DEF #	Length (bp)	Organism	Accession	Description	Functional category	Tissue	*E* value	Maximum identity
*Class 1: transient-induced expression*								
**018**	324	*Zea mays*	AAD20307.1	Copia-type pol polyprotein	Unclassified	Leaf (3 days)	1.*E* − 46	94%
028	444	*Oryza sativa*	AAK13160.1	Putative esterase	Esterase	Leaf (5 days)	3.*E* − 28	84%
**093**	201	*Zea mays*	NP_001150949.1	bZIP transcription factor ABI5	Transcription	Leaf (5 days)	6.*E* − 07	100%
**096**	201	*Zea mays*	NP_001150949.1	bZIP transcription factor ABI5	Transcription	Leaf (3 days)	6.*E* − 07	100%
116	198	*Arabidopsis lyrata*	XP_002866900.1	DNAJ heat shock family protein	Genetic information processing	Leaf (3 days)	3.*E* − 14	79%
117	193	*Glycine max*	BAG09374.1	Peroxisomal hydroxypyruvate reductase	Metabolism	Leaf (3 days)	4.*E* − 13	88%
206	424	*Oryza sativa*	BAA24573.1	DNA polymerase alpha catalytic subunit	Metabolism	Leaf (3 days)	5.*E* − 20	84%
211	440	*Arachis hypogaea*	AEL30371.1	TIR-NBS-LRR type disease resistance protein	Organismal systems	Root (3 days)	9.*E* − 20	65%
234	492	*Zea mays*	ACR36983.1	Unknown	Unclassified	Root (3 days)	1.*E* − 11	64%
332	350	*Zea mays*	ACG29577.1	Cytochrome P450 CYP78A55	Metabolism	Leaf (3 days)	7.*E* − 03	74%

*Class 2: rapidly switched off*								
002	675	*Zea mays*	NP_001105935.1	Elongation factor alpha8	Genetic information processing	Leaf	2.*E* − 77	99%
145	310	*Arabidopsis thaliana*	NP_567433.1	SRPBCC ligand-binding domain-containing protein	Unclassified	Root	1.*E* − 24	51%
161	316	*Zea mays*	NP_001183823.1	Hypothetical protein LOC100502416	Unclassified	Leaf	9.*E* − 10	59%
188	398	*Zea mays*	NP_001148699.1	Tyrosyl-tRNA synthetase	Genetic information processing	Root	2.*E* − 04	100%
257	360	*Zea mays*	ACG45582.1	Histone-lysine N-methyltransferase, H3 lysine-9 specific SUVH1	Metabolism	Leaf	3.*E* − 55	88%

*Class 3: gradually switched-off expression*								
**005**	427	*Zea mays*	NP_001150729.1	Aspartic proteinase oryzasin-1	Signal transduction	Leaf	3.*E* − 66	99%
006	356	*Arabidopsis lyrata*	XP_002877001.1	Hydroxyproline-rich glycoprotein family protein	Metabolism	Leaf	2.*E* − 06	40%
026	696	*Arabidopsis thaliana*	AAM64166.1	Cleavage stimulation factor 77	Unclassified	Leaf	5.*E* − 42	67%
031	341	*Zea mays*	AAP94585.1	Putative gag-pol precursor	Unclassified	Leaf	3.*E* − 38	82%
076	353	*Oryza sativa*	ABA94145.1	Retrotransposon protein	Unclassified	Leaf	2.*E* − 45	83%
085	331	*Candidatus Liberibacter americanus*	ACD87749.1	Glycerol kinase	Metabolism	Leaf	1.*E* − 36	76%
**094**	201	*Zea mays*	NP_001150949.1	bZIP transcription factor ABI5	Transcription	Leaf	4.*E* − 17	100%
**243**	307	*Zea mays*	AAD20307.1	Copia-type pol polyprotein	Unclassified	Root	2.*E* − 46	89%
**262**	209	*Oryza sativa*	AAK13589.1	rRNA intron-encoded homing endonuclease	Genetic information processing	Leaf	2.*E* − 12	88%

*Class 4: induced expression*								
044	346	*Zea mays*	ACG29272.1	Hypothetical protein	Unclassified	Root	8.*E* − 05	73%
104	425	*Oryza sativa Japonica group*	ABF97883.1	Retrotransposon protein	Unclassified	Root	9.*E* − 23	77%
**115**	209	*Zea mays*	NP_043052.1	Photosystem II protein H	Photosynthesis	Leaf	1.*E* − 05	84%
179	240	*Zea mays*	BAA22440.1	Fatty acid desaturase	Metabolism	Leaf	1.*E* − 01	100%
194	273	*Litchichinensis*	ADZ76112.1	Actin	Metabolism	Leaf	7.*E* − 47	98%
202	239	*Zea mays*	ACG41852.1	Pyruvate decarboxylase isozyme 1	Metabolism	Root	1.*E* − 48	100%
221	477	*Oryza sativa*	ABF95989.1	Retrotransposon protein, putative, Ty3-gypsy subclass, expressed	Unclassified	Root	9.*E* − 45	60%
**267**	209	*Oryza sativa*	AAK13589.1	rRNA intron-encoded homing endonuclease	Genetic information processing	Root	2.*E* − 12	88%

*Class 5: switched-off–induced expression*								
**013**	427	*Zea mays*	NP_001150729.1	Aspartic proteinase oryzasin-1	Signal transduction	Root	7.*E* − 49	99%
054	494	*Zea mays*	ACG34068.1	KOB1	Unclassified	Root	3.*E* − 38	72%
173	142	*Photorhabdus asymbiotica*	YP_003039426.1	Exonuclease V subunit gamma	Genetic information processing	Root	1.*E* + 00	72%
223	343	*Oryza sativa*	AAL47203.1	Chromatin-remodeling factor CHD3	Transcription	Root	7.*E* − 37	60%
239	382	*Zea mays*	AAL66757.1	Putative polyprotein	Unclassified	Root	2.*E* − 59	88%
337	367	*Zea mays*	NP_001141422.1	Protease Prl C candidate 1	Unclassified	Root	8.*E* − 21	91%

*Class 7: down expression*								
**101**	201	Zea mays	NP_001150949.1	bZIP transcription factor ABI5	Transcription	Root	6.*E* − 07	100%

*Class 8: down-up expression*								
062	401	*Triticum aestivum*	CBH32516.1	Alpha-glucan phosphorylase	Metabolism	Leaf	1.*E* − 62	92%
**181**	314	*Zea mays*	NP_001146965.1	Vacuolar ATPase subunit H protein	Transport	Root	7.*E* − 51	97%
**235**	471	*Arabidopsis thaliana*	NP_565520.1	Violaxanthin de-epoxidase-related	Metabolism	Root	5.*E* − 40	50%

*Class 10: up expression*								
079	352	*Oryza sativa*	AAQ56518.1	Putative polyprotein	Unclassified	Root	1.*E* − 45	77%
120	279	*Arabidopsis lyrata*	XP_002893119.1	Tubulin family protein	Transport	Root	2.*E* − 36	67%
189	250	*Zea mays*	ABF67926.1	Milt 1 A putative polyprotein	Unclassified	Root	2.*E* − 39	91%
205	447	*Nicotiana tabacum*	AAF33670.1	Cyclic nucleotide-gated calmodulin-binding ion channel	Signal transduction	Leaf	3.*E* − 41	56%
293	364	*Zea mays*	AAL73444.1	Putative non-LTR retroelement reverse transcriptase	Genetic information processing	Root	2.*E* − 04	61%

*Class 11: up-down expression*								
112	279	*Populus trichocarpa*	XP_002329950.1	Tubulin gamma complex-associated protein	Signal transduction	Leaf	5.*E* − 42	78%
114	279	*Populus trichocarpa*	XP_002329950.1	Tubulin gamma complex-associated protein	Signal transduction	Leaf	1.*E* − 35	78%
**139**	209	*Zea mays*	NP_043052.1	Photosystem II protein H	Photosynthesis	Root	1.*E* − 05	84%
**176**	314	*Zea mays*	NP_001146965.1	Vacuolar ATPase subunit H protein	Transport	Leaf	6.*E* − 50	95%
186	412	*Zea mays*	AAN40029.1	Putative gag-pol precursor	Unclassified	Leaf	8.*E* − 59	87%
**231**	471	*Arabidopsis thaliana*	NP_565520.1	Violaxanthin de-epoxidase-related	Metabolism	Leaf	5.*E* − 40	50%

*Class 12: up-down-up expression*								
**268**	209	*Oryza sativa*	AAK13589.1	rRNA intron-encoded homing endonuclease	Genetic information processing	Root	2.*E* − 12	88%
334	425	*Oryza sativa*	ABA99740.2	Retrotransposon protein, putative, unclassified	Unclassified	Root	9.*E* − 31	43%

*Class 13: induced–switched-off expression*								
050	401	*Zea mays*	ACG34068.1	KOB1	Unclassified	Leaf	1.*E* − 51	100%
075	407	*Zea mays*	ABF67934.1	Opie3 pol polyprotein	Unclassified	Leaf	2.*E* − 24	70%
172	284	*Arabidopsis thaliana*	NP_564010.1	Uridylyltransferase-related protein	Transferase	Leaf	9.*E* − 07	74%
209	489	*Glycine max*	ABB00038.1	Reverse transcriptase family member	Genetic information processing	Root	1.*E* − 52	51%
214	479	*Gossypium hirsutum*	AAP43917.1	Integrase	Integrase	Leaf	3.*E* − 52	77%
220	477	*Zea mays*	AAL59229.1	Gag-pol	Unclassified	Root	5.*E* − 56	62%

## Data Availability

The data used to support the findings of this study are available from the corresponding author upon request.
